# The effects of curcumin and a modified curcumin formulation on serum Cholesteryl Ester Transfer Protein concentrations in patients with metabolic syndrome: A randomized, placebo-controlled clinical trial

**Published:** 2018

**Authors:** Ali Javandoost, Asma Afshari, Maryam Saberi-Karimian, Amirhosein Sahebkar, Hamideh Safarian, Maliheh Moammeri, Behdokht Fathi Dizaji, Shima Tavalaei, Gordon A. Ferns, Alireza Pasdar, Seyed Mohammad Reza Parizadeh, Majid Ghayour-Mobarhan

**Affiliations:** 1 *Metabolic Syndrome Research Center, Department of Biochemistry, Faculty of Medicine, Mashhad University of Medical Sciences, Mashhad, Iran*; 2 *Department of Nutrition, Faculty of Medicine, Mashhad University of Medical Sciences, Mashhad, Iran*; 3 *Student Research Committee, Department* *of Modern Sciences* *and Technologies, Faculty of Medicine, Mashhad* *University of Medical Sciences, Mashhad, Iran*; 4 *Biotechnology Research Center, Mashhad University of Medical Sciences, Mashhad, Iran*; 5 *Department of Genetic, Faculty of Medicine, Mashhad University of Medical * *Sciences, Mashhad, Iran*; 6 *Brighton & Sussex Medical School, * *Division of Medical Education, Mayfield House, Brighton, Sussex, BN1 9PH UK*; 7 *Division of Applied Medicine, Medical School, University of Aberdeen, Foresterhill, Aberdeen, AB25 2ZD, UK*; 8 *Cardiovascular Research Center, School of Medicine, * *Mashhad University of Medical Sciences, Mashhad, Ira* *n*

**Keywords:** Metabolic syndrome, HDL-C, CETP, Curcumin, Atherogenesis

## Abstract

**Objective:**

Cholesteryl Ester Transfer Protein (CETP) mediates the transfer of cholesteryl ester from HDL-C to LDL-C and VLDL-C. The aim of the present trial was to evaluate the effect of curcumin and its modified formulation on serum CETP concentrations in patients with metabolic syndrome.

**Materials and Methods:**

Participants were randomly allocated to one of three groups of 40 subjects receiving either unmodified curcumin or its phospholipid complex or placebo. Lipid profile and plasma CETP were measured at the start and six weeks after initiation of the treatment. The normality of data distribution was assessed by Kolmogorov-Smirnov test. Wilcoxon test was used for comparing the data before and after the intervention. The percent changes of CETP and biochemical factors among the three groups were compared using Kruskal-Wallis test.

**Results:**

Serum CETP levels were not significantly altered among patients receiving curcumin.

**Conclusion:**

Curcumin and its complex had no significant effect on serum CETP concentrations.

## Introduction

Metabolic syndrome is characterized by a cluster of metabolic abnormalities leading to an increased risk of cardiovascular diseases (CVD) and diabetes mellitus (DM) )Albert KGM et al.,2005(. Dyslipidaemia (high serum levels of triglyceride, cholesterol, low-density lipoprotein (LDL) cholesterol, or low serum levels of high-density lipoprotein (HDL) cholesterol) is a common risk factor contributing to atherosclerosis )Chapman et al., 2011 ▶(. HDL has been regarded as a protective factor against atherosclerosis by virtue of the mechanism of reverse cholesterol transport (RCT) )Fisher et al., 2012 ▶ (. Cholesteryl Ester Transfer Protein (CETP) is involved in transferring cholesterol esters from HDL to particles with higher fat content like LDL and very low density lipoprotein (VLDL) )Curb et al., 2004 ▶;Sandhofer et al., 2006 ▶(. Therefore, modifying the activity of these proteins may have an impact on atherosclerosis and metabolic syndrome )Sandhofer et al., 2006 ▶).

Phytochemicals such as polyphenols are potentially important compounds for the prevention of CVD (Upadhyay and Dixit, 2015 ▶). Curcumin is a natural phytochemical compound found in turmeric, which comes from the *Curcuma longa* (Aggarwal, 2010 ▶). There has been growing evidence regarding the effects of curcumin on the treatment of conditions including cancer, HIV, obesity and CVD (Epstein et al.,2010 ▶; Shehzad and Lee, 2010 ▶; Maheshwari et al., 2006 ▶; Anand et al., 2008 ▶; Julie and Jurenka, 2009 ▶). 

 Polyphenols have some beneficial effects including raising HDL, lowering blood pressure and reducing weight, via several possible mechanisms, including their effects on CETP (Aggarwal et al., 2005 ▶; Sahebkar, 2014 ▶). Since curcumin is not well absorbed via the oral route, other more bioavailable forms of curcumin have been developed; for example, the phospholipid complex of curcumin as well as micellar formulations have been prepared to improve curcumin kinetics (Marczylo et al., 2007 ▶; Kidd, 2009 ▶). Consequently, this study was conducted in order to compare the effect of curcumin and complex curcumin on CETP level among patients with metabolic syndrome.

## Materials and Methods

**Study Participants**

This study was a randomized, double- blind clinical trial which was approved by the Ethics Committee and licensing authorities of the Nutrition Clinic of Qaem Teaching Hospital, Mashhad, Iran. This research was carried out among subjectsof18 to 65 years old who were diagnosed with metabolic syndrome, according to the International Diabetes Federation (IDF- 2006) criteria. Exclusion criteria were pregnancy, lactation, systemic diseases including lupus and renal disorders as well as the use of cholesterol-lowering or other lipid-lowering drugs within the last 6 months. According to the changes in mean triglyceride before and after curcumin consumption among experimental and placebo groups, the sample size in each group was calculated as 35 subjects withtype one error 0.05 and power of 80%. Considering 15% dropout in samples, the sample size in each group was estimated as 40 subjects. 

**Study design**

**Figure 1:**
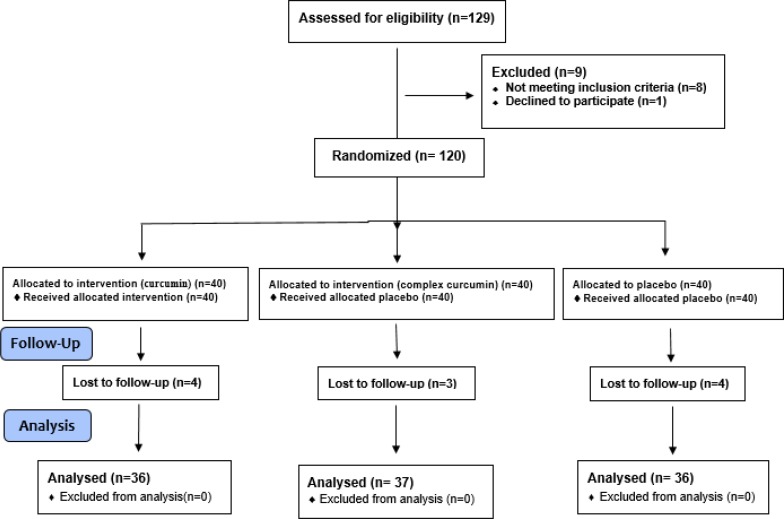
Flow chart of participants

Patients referring to the dietetic clinic at Qaem Hospital were sequentially assigned to one of the treatment protocols by the researchers one after another; the curcumin group, and the curcumin phospholipid complex group taking 500 mg curcumin twice a day and a placebo group (Figure. 1). Placebo tablets were similar in appearance, to curcumin and curcumin phospholipid complex tablets, but only contained starch and had an approved color. Curcumin and curcumin phospholipid complex were supplied by Indena S.p.A (Milan, Italy). 

The study protocol has been registered in the Iranian Registry of Clinical Trials (IRCT2014052014521N3) (Saberi-Karimian et al., 2018 ▶). 

After obtaining signed informed consent from participants and receiving approval from Ethics Committee of Mashhad University of Medical Sciences, Mashhad, Iran, anthropometric indices and blood pressure were measured and demographic characteristics, history of smoking, history of pharmaceutical medication and levels of physical activity were recorded.

During the study, all individuals received a similar diet and were visited twice during the six-week period. Anthropometric indices were measured using a standard scale (0.1 kg accuracy) and a stadiometer (0.1 cm accuracy). Patients were asked to stand without shoes with shoulders in contact with the wall. Waist circumference was measured at the midpoint between the last rib and the iliac crest while the hip circumference was measured at maximum hip circumference. 

Blood samples were obtained after 8-12 hr of fasting. After centrifugation, serum was separated and stored at - 80 ° C until analysis. Serum total cholesterol, LDL-Cholesterol, HDL-Cholesterol and triglyceride levels were measured by an auto-analyzer using commercial kits (Pars Azema Company, Teheran, Iran). Blood pressure (BP) was measured using Omron digital twice after 15 min rest in a sitting position.

**CETP measurement**

Before and after the intervention, serum CETP levels were measured using a Human enzyme immunoassay Kit (Cusabio biotech Inc., China) and an ELISA Plate Reader device (STAT FAX 2100, USA),in accordance with the guidelines of the kit, respectively, the detection range was 0.78-50 ng/ml and the sensitivity was 0.2 ng/ml. 

All measurements were made in duplicate, and the intra-assay and inter-assay coefficients of variation were reported to be <8%and <10%, respectively. The precision and accuracy of all methods used in this study were checked using commercially prepared control sera (Cusabio biotech Inc., China). 

**Statistical analysis**

Data were analyzed using SPSS version 16 software. A p-value<0.05 was considered statistically significant. Qualitative data including gender was compared among the three groups using Chi-square test. The normality of data distribution was assessed by Kolmogorov-Smirnov test. The non-normal data was compared using Wilcoxon (before and after intervention), one way ANOVA or Kruskal-Wallis tests (among groups). The percentage changes in CETP and biochemical factors among the three groups were compared using Kruskal-Wallis and a Bonferroni correction was made *post-hoc*. 

## Results

Baseline demographic, clinical and biochemical characteristics of the patients in three groups are illustrated in Table 1. Only weight was significantly different among groups (p=0.01).

**Table 1:** Baseline demographic clinical and biochemical variables of participants in the three groups

**Variable**	**Study group**			**P-value**
	**Curcumin**	**Complex curcumin**	**Placebo**	
	**Mean±SD**	**Mean±SD**	**Mean±SD**	
**CETP (µg/ml)**	0.43±0.17	0.47±0.13	0.42±0.14	0.11
**Weight (kg)**	81.9±1.1	85.32±1.7	83.7±1.0	0.01
**BMI (kg/m** ^2^ **)**	31.3±3.3	31.8±5.4	32.2±5.2	0.18
**WC (cm)**	101.7±9	104.6±9.8	104±9.2	0.06
**Fat ** **)** **%)**	35.16±6.4	35.12±8.3	36.4±9.8	0.8
**FBG (mg/dl)**	101.06±25.3	99.25±24.1	96.72±18.17	0.47
**SBP (mmHg)**	121.1±12.0	124.00±9.8	122.80±7.8	0.35
**DBP (mmHg)**	82.38±10.46	86.52±10.58	83.88±9.31	0.78

WC, waist circumferences; FBG, fasting blood glucose; DBP; diastolic blood pressure, SBP; systolic blood pressure. Values are expressed as mean±SD.

As shown in Table. 2, CETP concentrations did not significantly change before and after the intervention in curcumin (p=0.3), and placebo (p=0.97) groups. A significant increase in the CETP concentrations was observed in the complex curcumin group alone (p=0.046).

**Table 2:** CETP and biochemical factors changes (delta, Δ) before and after intervention among the three groups

		**CETP** **(µg/ml)**	**Cholesterol** **(mg/dl)**	**TG** **(mg/dl)**	**HDL** **-C** **(mg/dl)**	**LDL** **-C** **(mg/dl)**	**FBG** **(mg/dl)**
**Curcumin**	**Before mean(SD)** *	0.43(0.17)	244.97(40.80)	169.73(88.70)	52.93(9.14)	157.34(35.10)	99.10(24.55)
	**After mean(SD)**	0.49(0.62)	246.67(48.90)	153.53(60.90)	51.97(7.39)	159.48(40.55)	101.83(15.63)
	**percent change(delta, Δ)**	15.29	1.10	-1.74	-0.65	1.74	6.22
	**p-value**	0.3	0.6	0.04	0.6	0.6	0.3
**Complex curcumin**	**Before mean(SD)** *	0.47(0.13)	232.41(49.24)	195.86(81.62)	48.83(11.07)	142.55(39.58)	98.50(21.80)
	**After mean(SD)**	0.65(0.39)	224.68(47.60)	189.86(75.99)	48.26(10.17)	137.30(40.23)	107.59(13.33)
	**percent change(delta, Δ)**	49.97	-2.94	1.49	-0.51	-3.72	12.86
	**p-value**	0.04	0.05	0.8	1	0.1	0.1
**Placebo**	**Before mean(SD)** *	0.43(0.15)	249.6(43.51)	187.9(51.64)	51.37(7.75)	163.41(37.22)	99.20(14.76)
	**After mean(SD)**	0.43(0.13)	224.9(40.59)	154.28(54.36)	48.74(7.89)	146.70(27.27)	102.15(16.34)
	**percent change(delta, Δ)**	7.19	-9.03	-15.22	-3.91	-7.48	3.17
	**p-value**	0.9	0.04	0.007	0.4	0.03	0.3
**P-value for between group changes** ***		0.1	0.04	0.09	0.5	0.07	0.4

* Data are presented as mean±standard deviation; the mean difference is significant at 0.05.

** Based on Wilcoxon test

*** Based on Kruskal-Wallis test

Based on the results shown in Table 2, there was no difference in the serum CETP changes among the groups (p=0.1). In addition, pair-wise comparisons using Bonferroni *post-hoc* tests showed that there were no significant differences in mean serum CETP concentrations between curcumin and complex curcumin groups (p=0.2), between curcumin and placebo groups (p=1) and between complex curcumin and placebo groups (p=0.9). The changes in the lipid profiles among different groups are also shown in Table 2. Among patients receiving curcumin, only triglyceride was significantly decreased (p=0.04), while patients who were receiving complex curcumin, only showed 2.94% cholesterol reduction, which showed a statistically significant difference (p=0.05). The cholesterol changes were significantly different between the groups (p=0.04) while, there were no significant differences in triglyceride (p=0.09), HDL (p=0.5), LDL (p=0.07) and fasting blood glucose (p=0.4) changes among groups.

Investigating the correlation between CETP changes and each of the clinical, anthropometric and biochemical factors, only indicated a significant negative correlation between serum HDL and CETP change among patients receiving complex curcumin (r=0.35, p=0.01). In addition, there were no significant interactions between CETP and biochemical factors on the effect of curcumin or modified curcumin (Table 3).

**Table 3:** Correlations between curcumin changes (delta, Δ) and biochemical factors among the three groups.

**Variables**	**CETP changes** **(delta, Δ)**						p-value for interactions
	**curcumin**		**Complex curcumin**		**placebo**		
	**Correlation coefficient**	**p-Value**	**Correlation coefficient**	**p-Value**	**Correlation coefficient**	**p-Value**	
**FBG (mg/dl)**	0.17	0.24	0.10	0.49	-0.001	0.95	0.2
**TG (mg/dl)**	0.06	0.63	0.03	0.80	0.02	0.88	0.2
**Cholesterol ** **(mg/dl)**	0.10	0.46	-0.38	0.09	0.08	0.57	0.1
**HDL-C (mg/dl)**	0.11	0.45	-0.35	0.01	0.24	0.11	0.8
**LDL-C (mg/dl)**	0.09	0.52	-0.20	0.18	0.01	0.92	0.3

HDL-C, high-density lipoprotein cholesterol; LDL-C, low-density lipoprotein cholesterol; FBG, fasting blood glucose.

## Discussion

There is a close relationship between metabolic syndrome and risk of cardiovascular diseases (Tavil et al., 2007 ▶); hence, it is important to determine factors that affect the metabolic and clinical components of metabolic syndrome in the prevention of deaths due to cardiovascular diseases (Ritchie S and Connell, 2007 ▶). 

Since few studies have investigated the influence of curcumin on human CETP, in this study, we assessed the impact of curcumin and its modified formulation (phospholipid) on CETP and lipid profile. Phosphatidylcholine complex of curcumin can enter the lipophilic cell membranes, thus increasing the bioavailability of curcumin. It has been proven that plasma concentration peak for phosphatidylcholine complex of curcumin, is five times higher than that of simple curcumin, in rats (Marczylo et al., 2007 ▶).

In the current trial, although some differences were observed between the effects of plain curcumin, curcumin complex and placebo on CETP, only the patients who received complex curcumin showed statistically significant increases in the concentration of CETP. However, these changes were not significantly different from those observed in the other groups indicating no influence of curcumin on the CETP. Elseweidy et al. (2015) ▶ reported the effect of curcumin on CETP in rabbits, finding significant differences between the groups taking curcumin and placebo (Elseweidy et al., 2015 ▶). However, such response might be due to an atherogenic diet which had been used for the study groups making them susceptible to treatment. Moreover, study participants were treated for only six weeks while longer-term treatment might significantly affect metabolic factors such as fat, LDL-C and TG as well as CETP. 

Shin et al. (2011) ▶ also studied the effect of the natural dietary compound curcumin on atherosclerosis in mice based on plasma and hepatic lipid metabolism. A high-cholesterol diet was given to the mice and they were treated with curcumin, lovastatin or control for 18 weeks. Curcumin lowered plasma cholesterol, triglycerides, LDL cholesterol and CETP activity and increased plasma HDL cholesterol similar to lovastatin (Shin et al., 2011 ▶). The reduced CETP activity was reported to be due to the changes in plasma cholesterol, independent of LDL receptors and direct inhibitory activity of curcumin on CETP remained unclear. The prolonged duration of this study and high-cholesterol diet used, can be considered the main differences between the two trials. 

However, non- significant interactions between CETP and biochemical factors in this study, indicate that some changes observed in these factors are not in keeping with CETP changes. It might be in contrast to the hypothesis stating that the effects of curcumin on serum lipids are associated with CETP. 

In spite of non-significant effect of curcumin on CETP, the inverse association between CETP and HDL-C should not be ignored. Results of different studies on various CETP inhibitors confirm such negative correlations. Ghatreh-Samani et al. (2012 ▶) showed that -629 C/A polymorphism in the promoter region of the *CETP* gene could decrease 50% of CETP protein and increase the HDL-C. In addition, inhibiting the CETP activity by statins administration leads to 5 to 15% increase in HDL-C concentration (Ghatreh-Samani et al.,2012 ▶). Davidson (2012) ▶ showed that CETP decreased following an increase in HDL-C by administrating a CETP inhibitor, dalcetrapib (Davidson, 2012 ▶). All of these results are in keeping with those observed in the current study.

It should be noted that most of the investigated serum lipid concentrations as well as blood glucose were not significantly changed following treatment with curcumin or curcumin complex. The exception was cholesterol which significantly reduced in the group treated with curcumin complex. Rahimi et al. (2016) ▶ compared serum levels of TC, TG, LDL-C, and HDL-C, before and after the treatment with nano-curcumin and found significant differences for each subject (Rahimi et al., 2016 ▶).

In conclusion, our study did not find evidence showing the effectiveness of curcumin in modification of serum CETP levels. Further studies are suggested to investigate these effects during longer periods. 
